# The Melanocortin MC5R as a New Target for Treatment of High Glucose-Induced Hypertrophy of the Cardiac H9c2 Cells

**DOI:** 10.3389/fphys.2018.01475

**Published:** 2018-10-26

**Authors:** Maria Consiglia Trotta, Rosa Maisto, Nicola Alessio, Anca Hermenean, Michele D’Amico, Clara Di Filippo

**Affiliations:** ^1^Department of Experimental Medicine, University of Campania “Luigi Vanvitelli”, Naples, Italy; ^2^Institute of Life Sciences, “Vasile Goldis” Western University of Arad, Arad, Romania

**Keywords:** cardiac hypertrophy, melanocortin 5 receptor agonism, glucose content, PI3K, GLUT1, GLUT4

## Abstract

The study explored the anti-hypertrophic effect of the melanocortin MC5R stimulation in H9c2 cardiac myocytes exposed to high glucose. This has been done by using α-MSH and selective MC5R agonists and assessing the expression of GLUT4 and GLUT1 transporters, miR-133 and urotensin receptor levels as a marker of cardiac hypertrophy. The study shows for the first time an up-regulation of MC5R expression levels in H9c2 cardiomyocytes exposed to high glucose medium (33 mM D-glucose) for 48 h, compared to cells grown in normal glucose medium (5.5 mM D-glucose). Moreover, H9c2 cells exposed to high glucose showed a significant reduction in cell viability (-40%), a significant increase in total protein per cell number (+109%), and an increase of the urotensin receptor expression levels as an evidence of cells hypertrophy. The pharmacological stimulation of MC5R with α-MSH (90 pM)of the high glucose exposed H9c2 cells increased the cell survival (+50,8%) and reduced the total protein per cell number (-28,2%) with respect to high glucose alone, confirming a reduction of the hypertrophic state as per cell area measurement. Similarly, PG-901 (selective agonist, 10^-10^ M) significantly increased cell viability (+61,0 %) and reduced total protein per cell number (-40,2%), compared to cells exposed to high glucose alone. Interestingly, the MC5R agonist reduced the GLUT1/GLUT4 glucose transporters ratio on the cell membranes exhibited by the hypertrophic H9c2 cells and increased the intracellular PI3K activity, mediated by a decrease of the levels of the miRNA miR-133a. The beneficial effects of MC5R agonism on the cardiac hypertrophy caused by high glucose was also observed also by echocardiographic evaluations of rats made diabetics with streptozotocin (65 mg/kg i.p.). Therefore, the melanocortin MC5R could be a new target for the treatment of high glucose-induced hypertrophy of the cardiac H9c2 cells.

## Introduction

Cardiac hypertrophy is caused by an increased glucose uptake into the cardiac myocytes that determines a high glucose-mediated oxidative stress into the cardiomyocytes (Kagaya et al., 1990; Zhang et al., 1995; Leong et al., 2002; Nascimben et al., 2004; Wang et al., 2009; Han et al., 2015; Wei et al., 2018). This increased glucose uptake is mostly due to an imbalance of the translocation of GLUT1 and GLUT4 glucose transporters from intracellular membranes to the cell surface of the myocytes with a GLUT1/GLUT4 ratio favoring GLUT1 (Slot et al., 1991; Abel et al., 1999; Paternostro et al., 1999; Tian et al., 2001; Kolwicz and Tian, 2011; Shao and Tian, 2015). In a normal adult heart GLUT4 is the primary glucose transporter translocating on plasma membrane after insulin stimulation, while the mediator of basal cardiac glucose uptake GLUT1 is downregulated after birth. Conversely, pathological hypertrophic condition links a GLUT4 depletion, resulting in a direct increase in GLUT1 levels (Slot et al., 1991; Abel et al., 1999; Paternostro et al., 1999; Tian et al., 2001; Kolwicz and Tian, 2011; Shao and Tian, 2015). Among these, GLUT4 expression and translocation is regulated by miR-133 both in skeletal muscle and in cardiac myocytes (Horie et al., 2009).

It is well known that regulation of the glucose homeostasis and insulin sensitivity involves the central melanocortin system, mostly through the hypothalamic proopiomelanocortin (POMC) which is well-established regulator of insulin secretion, glucose utilization, and glucose production. However, scant data exist about the role of peripheral melanocortin peptides or peptidomimetics in this regulation (Fan et al., 2000; Costa et al., 2006; Hill and Faulkner, 2017). Recently, it has been shown that peripheral α-melanocyte stimulating hormone (α-MSH) promotes glucose uptake in the skeletal muscle via melanocortin receptor 5 (MC5R) pathway (Enriori et al., 2016), suggesting a key role of this peptide and this receptor in the glucose transport and pathologies related to an altered glucose uptake. Interestingly, a recent human study showed that single-nucleotide polymorphism in the MC5R was associated with type 2 diabetes in obese subjects (Valli-Jaakola et al., 2008).

Therefore, in light of these evidences the aim of this study was to assess the anti-hypertrophic potential of the melanocortin MC5 receptor in H9c2 cardiomyocytes exposed to high glucose. Particularly, it has been investigated the phosphoinositide 3-kinase (PIK3) activity as a possible intracellular target of the MC5R stimulation. PI3K is a major player for mediating cardiac glucose since it is a regulator of glucose transporters (Egert et al., 1997; Vlavcheski et al., 2018), it is involved in reduction of cardiac hypertrophy (Weeks et al., 2017), and it is stimulated by the MC5R after α-MSH activation in HEK293 cells (Rodrigues et al., 2009).

## Materials and Methods

### Cell Culture and Treatments

Embryonic rat cardiac H9c2 (2-1) cells (ECACC, United Kingdom) were cultured in Dulbecco’s modified Eagle’s medium (DMEM; AU-L0101Aurogene, Italy), containing 5.5 mM D-glucose and supplemented with 10% Heat Inactivated Fetal Bovine Serum (AU-S181H Aurogene, Italy), 5% L-Glutamine (AU-X0550 Aurogene, Italy) and 5% Penicillin-Streptomycin Solution (AU-L0022 Aurogene, Italy), at 37°C under an atmosphere of 5% CO_2_ (Trotta et al., 2017). Then, H9c2 (2-1) cells were exposed to 5.5 mM D-glucose (Normal control group, NG); 5.5 mM D-glucose + Angiotensin II (1 μM; A9525 Sigma, Italy; Ang II group, positive control for cardiomyocytes hypertrophy) (Stuck et al., 2008); 33 mM D-glucose (A24940-01 Life Technologies, Italy; High glucose group, HG) (Wei et al., 2018); 33 mM D-glucose + α-MSH (90 pM; M4135 Sigma, Italy; HG + α-MSH group) (Enriori et al., 2016); 33 mM D-glucose + MC5R agonist PG-901 (10^-10^ M), dissolved in PBS (HG + PG-901 group) (Maisto et al., 2017); 33 mM D-glucose + MC5R antagonist PG-20N (130 nM), dissolved in PBS (HG + PG-20N group) (Rossi et al., 2016). H9c2 cardiomyocytes were stimulated with high glucose medium, with or without α-MSH,PG-901, and PG-20N treatment, for 48 h, being the 33 mM high glucose concentration reported to induce cardiac hypertrophy in H9c2 cardiomyocvtes after 2 days of exposure (Han et al., 2015; Zhong et al., 2015; Wei et al., 2018). Different cell numbers were used in the different experiments: for the viability assay, cells were seeded at 5 × 10^3^ cells/cm^2^ (Wei et al., 2018); the cells used for total RNA and protein detection were seeded at 1 × 10^6^ cells/cm^2^ (Han et al., 2015); for immunofluorescence assay, cells were seeded at 1 × 10^4^ cells/cm^2^ (Suhaeri et al., 2015); for isolation of membrane fractions cells were seeded at 4.1 × 10^3^ cells/cm^2^ (Yu et al., 2000). Three independent experiments were performed, each done in triplicate. H9c2 cell morphology was daily observed with optic microscope (Leica DMi1, Germany) and cell area was quantified using Image J software, by determining the average area per cell following a treatment and counting over 250 cells per well examined across triplicate wells (Wang et al., 2009).

### Anti-miR-133a Transfection

H9c2 cells were transfected with Anti-rno-miR-133a (MIN0000839 Qiagen, Italy) or negative control (1027271 Qiagen, Italy) using Lipofectamine 2000 reagent (11668-027 Life Technologies, Italy), according to the manufacturer’s protocol.

### Cell Viability Assay

H9c2 viability was measured by 3-(4,5-dimethylthiazol2-yl)-2,5-diphenyltetrazolium bromide (MTT)assay. 5 × 10^3^ cells/well were seeded in 96-well plates and treated as previously described. Briefly, MTT solution (1:10 in culture medium) was added to each well, incubated for 4 h and then removed. Each well was then washed for 20 min with isopropanol-HCl 0.2 N. Optical density (OD) values were measured at 570 nm using a 96-well plate reader (iMark, Bio-Rad Laboratories, Italy).

### MC5R mRNAs and miR-133a Levels Determination

For MC5R mRNAs determination levels, total RNA isolation was performed according the RNeasy Mini kit (74134 Qiagen, Italy), following the Purification of Total RNA from Animal Cells RNA concentration and purity were determined using a NanoDrop 2000c Spectrophotometer (Thermo Fisher Scientific, Italy). According to Siniscalco et al. (2013), 1 μg of RNA was reverse transcripted following the manufacturer’s protocol by using Superscript III reverse transcriptase system (4367659 Invitrogen, Italy) and oligo(dT)15. Real-time PCR was performed with Reddy Mix PCR Master Mix (AB-0575/DC/LD/B ThermoScientific, Italy), each reaction consisting in 1 μl of diluted cDNA, 22.5 μl of 1.19 ReddyMix PCR MasterMix, 1 μl of ddH_2_O and 1 μl of rat MC5R primer assay (QT01701920 Qiagen, Italy). The amplification profile used was the following: 95°C for 2 min; 35 cycles 94°C for 30 s, 55°C for 35 s, and 72°C for 65 s, followed by final elongation step at 72°C for 5 min. MC5R data were normalized relative to GAPDH and then used to calculate expression levels, according the 2^-ΔΔCt^ method.

For miR-133a determination levels, miRNAs isolation was performed according to the miRNeasy Mini kit (217004 Qiagen, Italy), following the supplementary protocol Purification of Total RNA, including Small RNAs, from Animal Cells. 5 μl of Syn-cel-miRNA-39 miScript miRNA Mimic 5 nM (MSY0000010 Qiagen, Italy) was spiked into each sample, before nucleic acid preparation, in order to monitor the miRNA recovery efficiency and to normalize miRNA expression in the Real-time PCR analysis. NanoDrop 2000c Spectrophotometer (Thermo Fisher Scientific, Italy) was used to determine RNA concentration and purity. Mature miRNA reverse transcription was performed according to the miScript II RT kit (218161 Qiagen, Italy). Then miR-133a expression levels were detected using the CFX96 Touch^TM^ Real-Time PCR Detection System (Bio-Rad Laboratories, Italy). Each reaction, carried out in triplicate, was set according the SYBR Green PCR Kit (218073 Qiagen, Italy) and by using specific miScript Primer Assays for miR-133a (MS00033208 Qiagen, Italy) and Syn-cel-miR-39 (MS00080247 Qiagen, Italy). Δ*C*t value for each miRNAas *C*t miRNA–*C*t Syn-cel-miR-39 was calculate to perform the relative quantization of miRNA expression; fold change was then obtained as 2^-ΔΔCt^. *P*-values are calculated with a Student’s *t*-test of the replicate 2^-ΔΔCt^ values for each miRNA in the different groups. A *P*-value less than 0.05 was considered significant.

### Immunocytochemistry

H9c2 cells were fixated with 4% paraformaldehyde, washed with PBS (AU-L0615 Aurogene, Italy) and then incubated for 30 min in blocking solution (1% BSA in PBS), in order to inhibit non-specific antibody binding. The primary antibodies, diluted in PBS blocking buffer and incubated overnight at 4°C, were: anti-GPR14 for Urotensin II receptor detection (sc-28998 Santa Cruz, United States) (Johns et al., 2004; Wang et al., 2009; Wei et al., 2018) and anti-actin (a-5441 Sigma, Italy). Specific antigens in each side were located using a Fluorescein Isothiocyanate (FITC) – conjugated anti-rabbit (GTXRB-003-D488N Immunoreagents, United States) and Tetramethylrhodamine (TRITC) – conjugated anti-mouse (GTXMU-003-D594N Immunoreagents, United States) secondary antibodies. H9c2 cells were counterstained with pentahydrate bisbenzimide (Hoechst 33258 Sigma, Italy) and then mounted with mounting medium (90% glycerol in PBS). Immunofluorescence images, obtained from the observation at a fluorescence microscope (Leica, Germany) and at a fluorescence confocal microscope (LSM 710 Zeiss, Germany), were analyzed with Leica FW4000 (Leica, Germany) and with Zen Zeiss (Zeiss, Germany) softwares. An observer blind to the treatment performed the labeling quantization for each microscope field, the percentage of positive cells was calculated by the number of labeled positive cells of 300 cells in four different microscope fields. In order to avoid overcounting cells, only bisbenzimide counterstained cells were considered as positive profiles, performing on each digitized image the cell positive profile quantization. Data are reported as the intensity means ± SEM of the percentages of positive cells / total cells counted in each analyzed field for each treatment. Three independent experiments were performed, each done in triplicate.

### Cell Lysate Preparation for Protein Quantization

Cells were washed with cold phosphate-buffered saline (PBS; AU-L0615 Aurogene, Italy); then 150 μl of cold RIPA lysis buffer (R0278 Sigma-Aldrich, United States) supplemented with a complete protease inhibitor cocktail (11873580001 Roche, United States) was added to each well. Lysates were collected and then centrifugated at 12,000 rpm for 10 min at 4°C. Total protein concentration in supernatants was measured using the Bio-Rad Protein Assay (500-0006 Bio-Rad Laboratories, Italy) and used for the determination of hypertrophy marker as total protein/viable cell number at direct cell counting (Wang et al., 2009), as well as for Western Blotting and ELISA assays.

### MC5R, Urotensin II Receptor and K_IR_6.1 Protein Levels Assessment

Western Blotting assay was performed in a 12% PAGE separation gel, electro-transferring 30 μg of protein sample onto a PVDF membrane (IPFL10100 Merck Millipore, Italy), blocked for 1 h at room temperature with 5% non-fat dry milk (EMR180500 Euroclone, Italy). Then blots were incubated over-night with the following specific primary antibodies: anti-MC5R (sc-28994 Santa Cruz, United States), anti-GPR14 for Urotensin II receptor detection (sc-288998 Santa Cruz, United States), anti K_IR_ 6.1 (P0874 Sigma, Italy), and anti-actin (sc-8432 Santa Cruz, United States). This step was followed by incubation for 1 h at room temperature with horseradish peroxidase-conjugated secondary anti-rabbit (sc-2004 Santa Cruz, United States) or anti-mouse (sc-2005, Santa Cruz, United States) antibodies. The signal was expressed as Densitometric Units (D.U.).

### PI3K Activity Determination

PI3K activity measurement was performed by using the PI3K Activity ELISA (K-1000s Echelon, Italy). The activity assay was performed following the immunoprecipitation of PI3K from cells, as suggested by the manufacturer’s instructions.

### Plasma Membrane GLUT1 and GLUT4 Levels Determination

H9c2 plasma membrane-enriched fractions were obtained by subcellular fractionation according to Yu et al. (2000). GLUT1 and GLUT4 levels from these fractions were measured using ELISA assays, according to the manufacturer’s instructions (MBS720405 and MBS451402 My BioSource, United Kingdom).

### Adenosine Triphosphate (ATP) Levels Determination

Rat ATP levels as marker of intracellular glucose content (Kuznetsov et al., 2015) were assayed in cell lysates by using Rat ATP Elisa kit (E02A0038BlueGene Biotech, China) according to the manufacturer’s protocol.

### Statistical Analysis

The results are presented as mean ± S.E.M. of three independent experiments, performing the triplicate of all the treatments in a single experiment. Statistical significance was determined using ANOVA followed by Bonferroni’s test. A *P*-value less than 0,05 was considered significant to reject the null hypothesis.

### *In vivo* Proof of Concept

To confirm the role of MC5R agonism in modulating cardiac hypertrophy induced by high-glucose exposure, we translated the *in vitro* experiments in a setting of *in vivo* ones, by investigating the effects of α-MSH and PG-901 in diabetic Sprague-Dawley rats. Male Sprague-Dawley rats (8 weeks of age), housed in a 12-h light/dark cycle animal room and fed with a standard chow diet and tap water *ad libitum*, were randomly divided into the following four groups (*n* = 5 for each group): (i) non-diabetic rats (CTRL); (ii) STZ-diabetic rats (STZ); (iii) STZ treated with α-MSH (STZ + α-MSH); and (iv) STZ treated with PG-901 (STZ + PG-901). Diabetes was induced in animals by a single intraperitoneal injection of 70 mg/kg STZ in 10 mM citrate buffer (pH 4.5; Sigma Chemical Co., United States) and 15 h later, human regular insulin (1.5 ± 0.5 units/day) was administered intraperitoneally yielding blood glucose levels of ∼22 mmol/l for 8 days (Di Filippo et al., 2005). Blood glucose greater than 300 mg/dL were verified 1 week after the STZ injection (Glucometer Elite XL; Bayer Co., Elkhart, IN, United States), in order to confirm diabetes development (Di Filippo et al., 2016). Then, diabetic rats received weekly intraperitoneal injections of 500 μg/kg α-MSH (Forslin Aronsson et al., 2007) (M4135 Sigma, Italy) or 50 – 500 – 5000 μg/kg PG-901. Animals were treated for 3 weeks after diabetes confirmation, and blood glucose levels were checked intermittently throughout the study to confirm diabetes maintenance. After the 3-week treatments, transthoracic echocardiography (Visualsonics Vevo 2100, Canada) was performed according to Di Filippo et al. (2014), using a 10–14 MHz linear transducer to obtain the images for the measurement of morphometric parameters, based on the average of three consecutive cardiac cycles for each rat. This study was carried out in accordance with to the guidelines of the Ethic Committee for animal experiments at the University of the Studies of Campania “Luigi Vanvitelli.”

## Results

### High Glucose Exposure Increases MC5R Levels in H9c2 Cells

**FIGURE 1:**
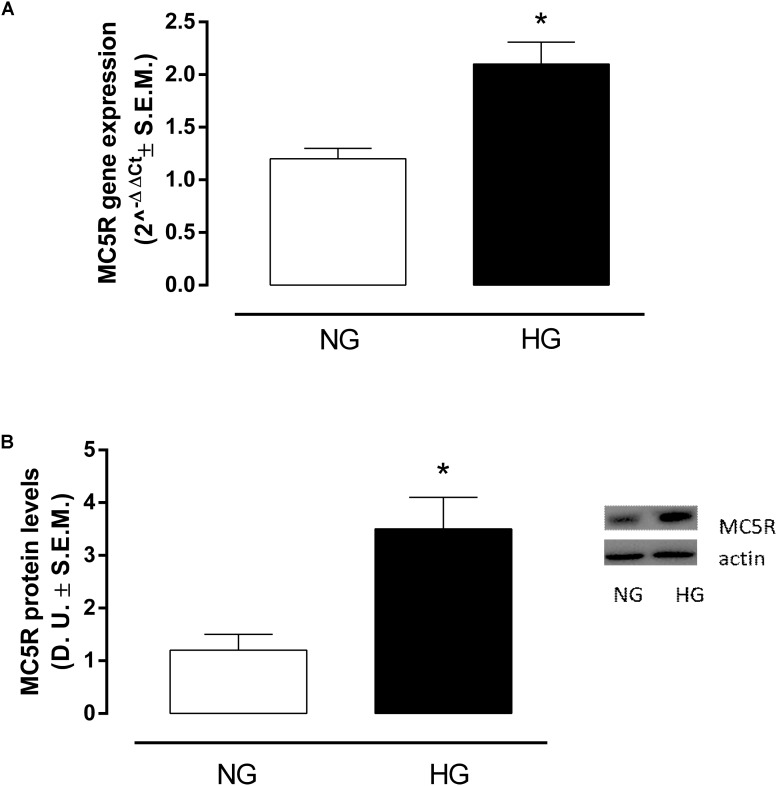
MC5R mRNA and protein levels. **(A)** RT-PCR analysis showed a significant up-regulation of MC5R in H9c2 cells exposed to high glucose (33 mM D-glucose) compared to cardiomyocytes exposed to normal glucose (5.5 mM D-glucose). **(B)** The significantMC5Rup-regulation in HG group was confirmed also by detection of MC5R protein levels by Western Blotting assay. Values are expressed as mean of 2^-ΔΔCt^ or D.U. ± S.E.M. of *n* = 9 values, obtained from the triplicates of three independent experiments. NG, normal glucose; HG, high glucose; D.U., Densitometric Units; ^∗^*P* < 0,01 vs. NG.

RT-PCR analysis showed that in H9c2 cells exposed to high glucose stimulus MC5R gene expression was significantly increased (*P* < 0,01 vs. NG) compared to control cells (Figure 1A). This was confirmed also by Western Blot Assay, showing a significant elevation of MC5R protein expression in H9c2 exposed to high glucose (*P* < 0,01 vs. NG), compared to control cells (Figure 1B).

### MC5R Agonism Reduces H9c2 Hypertrophy Induced by High Glucose, Increasing Cell Survival

**FIGURE 2:**
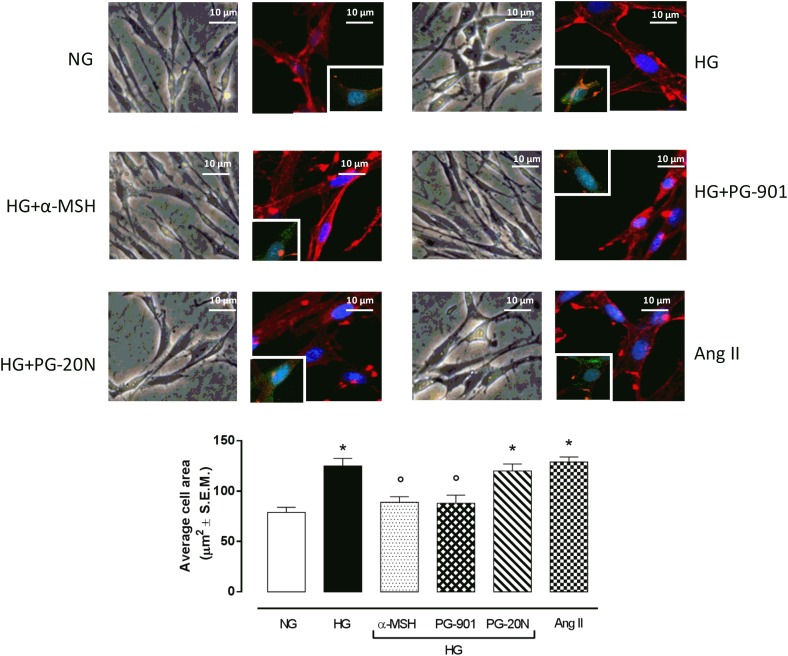
Cell size visualization and quantization. Representative optic microscope (40X) and immunofluorescence images (40X; in blue Hoechst for nucleus and in red actin for cytoskeleton labeling) show that H9c2 cells exposed to high glucose (33 mM D-glucose) exhibited an evident increase in cell area compared to cells exposed to normal glucose (5.5 mM D-glucose). MC5R agonism with α-MSH (90 pM) and PG-901 (10^-10^ M) treatment significantly decreased cell area in H9c2 cells exposed to high glucose. Conversely, H9c2 cells treated with MC5R antagonist PG-20N and exposed to high glucose showed a cell area similar to HG cells. Also representative images of cell nucleus (blue), actin (red), and urotensin II receptor (anti-GPR14, green) co-localization are shown, evidencing that the increased cell area in H9c2 cells exposed to high glucose is paralleled by a high Urotensin II receptor labeling. This is decreased by α-MSH (90 pM) and PG-901 (10^-10^ M) treatments. Cell area was calculating by analyzing 300 cells in four different microscope fields. Values are expressed as mean ± S.E.M. of *n* = 9 values, obtained from the triplicates of three independent experiments. NG, normal glucose; Ang II, angiotensin II; HG, high glucose; ^∗^*P* < 0,01 vs. NG; °*P* < 0,01 vs. HG.

**FIGURE 3:**
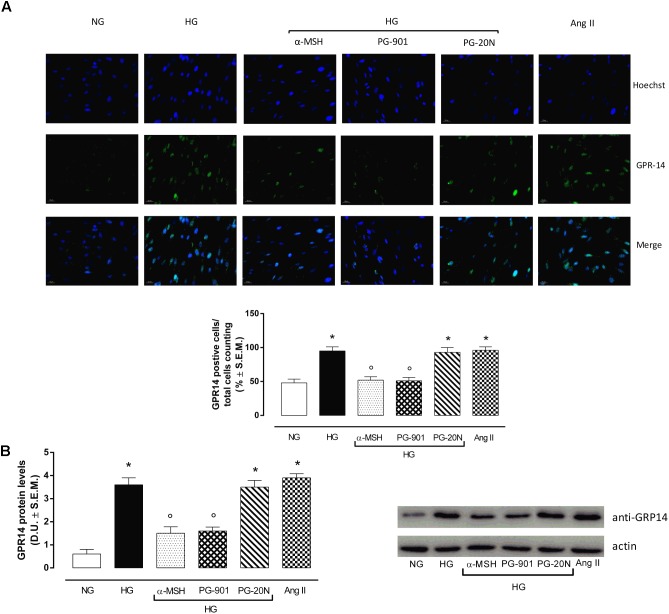
Urotensin II receptor immunocytochemistry and Western Blotting Assay. **(A)** Representative immunofluorescence images (20X; in blue Hoechst for nucleus and in green GRP14 for Urotensin II receptor labeling) show that H9c2 cells exposed to high glucose (33 mM D-glucose) exhibited an evident increase Urotensin II levels, highly expressed in hypertrophic cardiomyocytes compared to cells exposed to normal glucose (5.5 mM D-glucose). MC5R agonism with α-MSH (90 pM) and PG-901 (10^-10^ M) treatment significantly decreased Urotensin II labeling in H9c2 cells exposed to high glucose. MC5R antagonist PG-20N treatment in H9c2 cells exposed to high glucose showed high Urotensin II levels. The percentage of positive cells was calculated by the number of labeled positive cells of 300 cells in four different microscope fields. **(B)** Western Blots analysis detecting GPR-14 protein levels confirmed the elevated protein expression of Urotensin II receptor in hypertrophic H9c2 cells; this was significantly reduced in H9c2 cells exposed to high glucose and treated with MCR5 agonists. Values are expressed as mean ± S.E.M. of *n* = 9 values, obtained from the triplicates of three independent experiments. NG, normal glucose; Ang II, angiotensin II; HG, high glucose; D.U., Densitometric Units; ^∗^*P* < 0,01 vs. NG; °*P* < 0,01 vs. HG.

**FIGURE 4:**
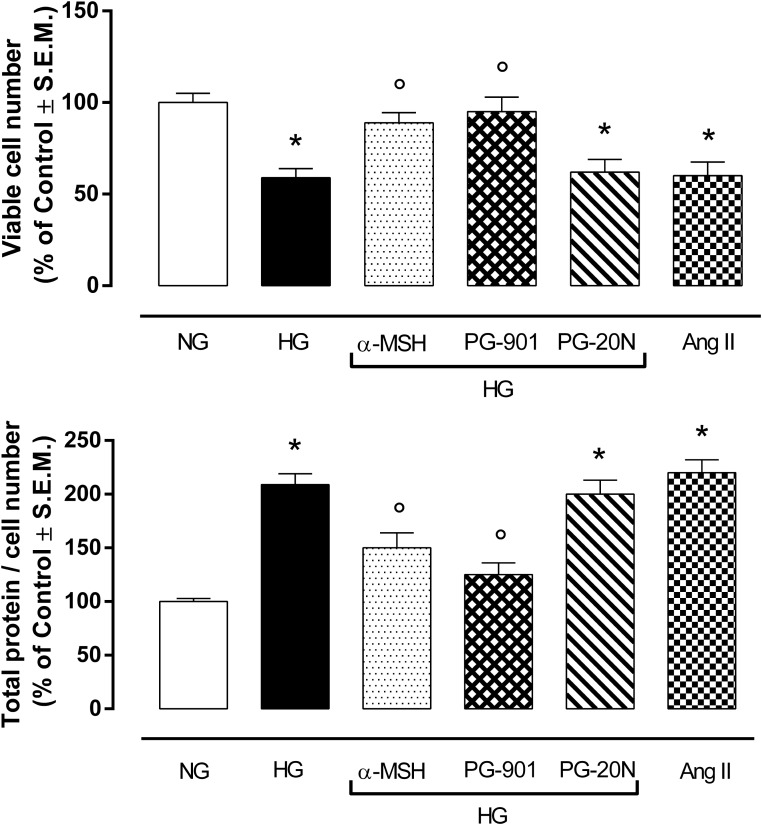
MTT assay and determination of total protein / viable cell number at direct cell counting. H9c2 cells exposed to high glucose (33 mM D-glucose) exhibited a significant decrease in cell viability and higher total protein / viable cell number ratio, as a marker of cardiac hypertrophy, compared to cell exposed to normal glucose (5.5 mM D-glucose). Agonism at MC5R with a-MSH (90 pM) and selectively with PG-901 (10^-10^ M) increased cell viability and decreased total protein / viable cell number ratio in cells exposed to high glucose. Cell viability values and total protein / cell number ratio exhibited by H9c2 cells exposed to high glucose were not affected by PG-20N MC5R antagonist (130 nM). Values are expressed as mean ± S.E.M. of *n* = 9 values, obtained from the triplicates of three independent experiments. NG, normal glucose; Ang II, angiotensin II; HG, high glucose; ^∗^*P* < 0,01 vs. NG; °*P* < 0,01 vs. HG.

H9c2 cell area quantization showed an evident increase in cell area in cardiomyocytes exposed to high glucose (HG) compared to cells exposed to normal glucose (NG; +58,2%, *P* < 0,01 vs. NG), indicating a hypertrophic condition (Figure 2). Agonism at MC5R with α-MSH (90 pM) and PG-901 (10^-10^ M) significantly reduced cell area in cells exposed to high glucose. This reduction was absent in H9c2 cells grown in high glucose and treated with MC5R antagonist (-28,8 and -29,6%, respectively, *P* < 0,01 vs. HG) PG-20N (130 nM) (Figure 2). GPR-14 immunofluorescence labeling confirmed the hypertrophy shown by H9c2 cells grown in HG compared to cells exposed to NG, showing a significant increase in GPR14levels (+111,1%, *P* < 0,01 vs. NG) (Figure 3). The high Urotensin II receptor levels in H9c2 cells exposed to high glucose were decreased by α-MSH (90 pM; -37,9%, *P* < 0,01 vs. HG) and PG-901 (10^-10^ M; -40,0%, *P* < 0,01 vs. HG), while they were not modified by PG-20N treatment (Figure 3A). Also GPR-14 Western Blotting Assay evidenced the same Urotensin II protein expression pattern in the different experimental settings (Figure 3B). These results were confirmed also by total protein/ cell number ratio, a sensible marker of hypertrophy (Figure 4): H9c2 cells exposed to high glucose showed a significant increase in total protein per cell number (+109%, *P* < 0,05 vs. NG), a significant reduction in cell viability (-40%, *P* < 0,01 vs. NG). α-MSH treatment reduced the total protein per cell number (-28,2%, *P* < 0,01 vs. HG) and increased cell survival (+50,8%, *P* < 0,01 vs. HG), confirming the reduction of hypertrophic state. Also PG-901 treatment reduced total protein per cell number (-40,2%, *P* < 0,01 vs. HG) and significantly increased cell viability (+61,0 %,*P* < 0,01 vs. HG) compared to cells exposed to high glucose. In contrast, an antagonism at MC5R with PG-20N did not produce effect on the parameters recorded in HG (Figure 4).

### Activation of MC5R Reduces Glucose Uptake and Increases K_IR_6.1 Expression in H9c2 Cells Exposed to High Glucose

**FIGURE 5:**
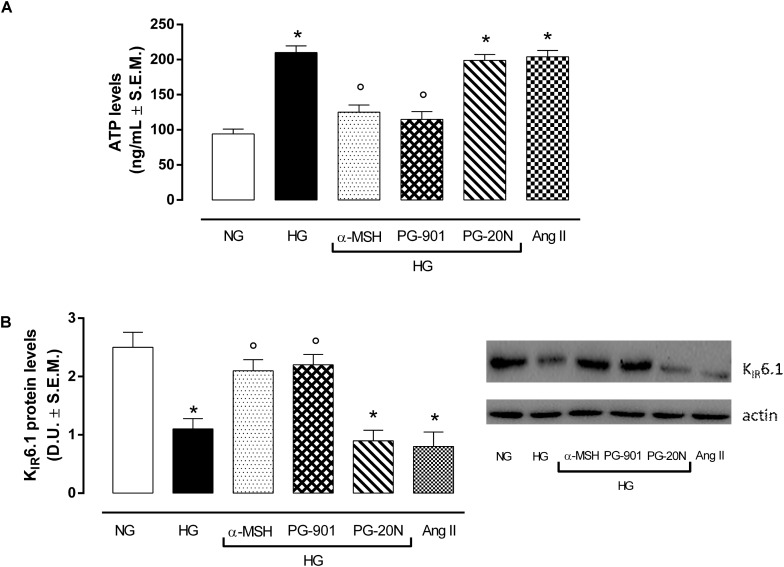
ATP levels as a marker of glucose content andKIR6.1 protein expression. **(A)** As expected, H9c2 cells exposed to high glucose (33 mM D-glucose) showed increased ATP levels compared to cell exposed to normal glucose (5.5 mM D-glucose). a-MSH (90 pM) and PG-901 (10^-10^ M) significantly decreased ATP levels, reducing the high cellular glucose uptake exhibited by HG cells. PG-20N antagonist did not modify ATP levels. **(B)** Western Blotting analysis of KIR6.1 protein levels showed a significant KIR6.1 down-regulation in H9c2 cells exposed to high glucose, probably due to high ATP intracellular content. The ATP levels reduction caused by MC5R agonists α-MSH and PG-901in H9c2 cells exposed to high glucose was paralleled by a significant increase in K_IR_6.1 protein expression. Values are expressed as mean ± S.E.M. of *n* = 9 values, obtained from the triplicates of three independent experiments. NG, normal glucose; Ang II, angiotensin II; HG, high glucose; D.U., Densitometric Units;^∗^*P* < 0,01 vs. NG; °*P* < 0,01 vs. HG.

In order to confirm the increase in glucose uptake, a feature of pathological cardiac hypertrophy, ATP levels were determined as marker of intracellular glucose content. As expected, H9c2 cells exposed to high glucose showed a significant increase in ATP level compared to cells exposed to normal glucose (+123,4%, *P* < 0,01 vs. NG). The agonism at MC5R with α-MSH and PG-901 significantly reduced cellular glucose uptake, as measured from ATP levels compared to HG cells (-40,5 and 45,2%, respectively, *P* < 0,01 vs. HG). The PG-20N antagonist did not lead to any change of cellular glucose content (Figure 5A). H9c2 cells exposed to high glucose were also characterized by a significant reduction in K_IR_6.1 protein levels (-56,0%, *P* < 0,01 vs. NG) compared to control cells, probably due to the high ATP intracellular levels. In contrast, the reduction in intracellular ATP content due to α-MSH and PG-901 MC5R agonism was paralleled by a significant increase in K_IR_6.1 protein expression (+90,9 and +100,0%, respectively, *P* < 0,01 vs. HG), compared to HG cells (Figure 5B).

### Reduction of miR-133a Levels by MC5R Agonists in H9c2 Cells Exposed to High Glucose Stimulus and Consequent PI3K Activation

**FIGURE 6:**
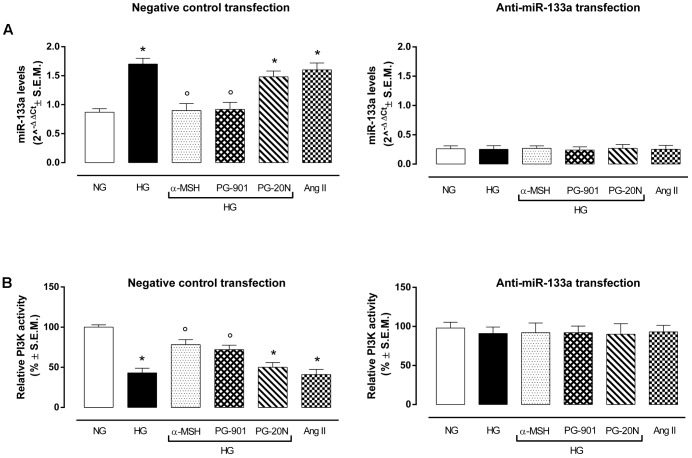
miR-133a levels and PI3K activity. **(A)** H9c2 cells exposed to high glucose (33 mM D-glucose) and transfected with negative control miRNA inhibitor showed increased miR-133a levels compared to cell exposed to normal glucose (5.5 mM D-glucose). a-MSH (90 pM) and PG-901 (10^-10^ M) significantly decreased miR-133a levels, while PG-20N antagonist didn’t modify miR-133a expression. As expected, miR-133a knockdown abolished this differential expression pattern in our experimental setting. Values are expressed as mean of 2^-ΔΔCt^ ± S.E.M. of *n* = 3 independent experiments, performing each treatment in triplicate in a single experiment. **(B)** Fitting with these evidences, PI3K activity was decreased in H9c2 cells exposed to high glucose, but it was significantly reverted by a-MSH (90 pM) and PG-901 (10^-10^ M).miR-133a knockdown reverted PI3K activity to values expressed by control cells in all the experimental conditions. Values are expressed as mean ± S.E.M. of *n* = 9 values, obtained from the triplicates of three independent experiments. NG, normal glucose; Ang II, angiotensin II; HG, high glucose; ^∗^*P* < 0,01 vs. NG; °*P* < 0,01 vs. HG.

qRT-PCR analysis showed an overexpression of miR-133a in H9c2 exposed to high glucose stimuli and transfected with negative control mina inhibitor, compared to control cells (+95,4%, *P* < 0,01 vs. NG) (Figure 6A). As expected, this was paralleled by a decreased PI3K activity in H9c2 cells exposed to HG (-57,0%, *P* < 0,01 vs. NG), being PI3K targeted by miR-133a (Figure 6B). Interestingly, α-MSH significantly reduced the miR-133a expression over by -47.0%, (*P* < 0,01 vs. HG) and consequently recovering PI3K activity (+82,1%, *P* < 0,01 vs. HG). These evidences on miR-133a and PI3K by α-MSH were copied by MC5R agonist PG-901 (-45,8 and +67,4%, respectively, *P* < 0,01 vs. HG). No sign of significative change was seen with PG-20N on the parameters recorded in HG (Figures 6A,B). H9c2 cells exposed to all experimental conditions and transfected with anti-miR-133a evidenced reduced miR-133a levels, as expected. These were paralleled by increased PI3Kactivity showed by H9c2 cells exposed to high glucose with or without MC5R agonists and antagonist (Figures 6A,B).

### MC5R Agonism Reduces the Elevated GLUT1/GLUT4 Ratio Induced by High Glucose in H9c2 Hypertrophy

**FIGURE 7:**
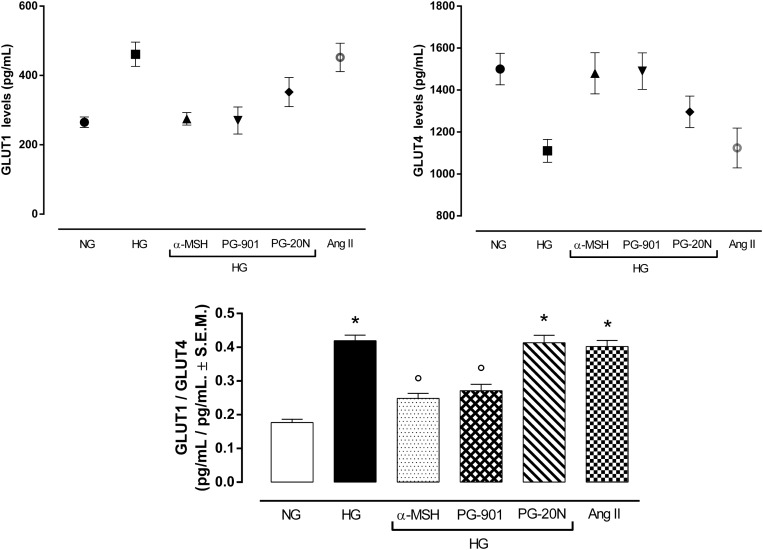
Plasma membrane GLUT1 and GLUT4 levels. In line with previous evidences, H9c2 cells exposed to high glucose (33 mM D-glucose) exhibited lower GLUT4 levels and increased GLUT1 levels in plasma membrane, resulting in a higher GLUT1/GLUT4 levels compared to cells exposed to normal glucose (5.5 mM D-glucose). a-MSH (90 pM) and PG-901 (10^-10^ M) significantly restored GLUT1/GLUT4 ratio, by increasing GLUT4 and consequently decreasing GLUT1 plasma membrane levels. PG-20N MC5R antagonist (130 nM) did not lead to any modification of the high GLUT1/GLUT4 ratio induced by high glucose. Values are expressed as mean ± S.E.M. of *n* = 9 values, obtained from the triplicates of three independent experiments. NG, normal glucose; Ang II, angiotensin II; HG, high glucose; ^∗^*P* < 0,01 vs. NG; °*P* < 0,01 vs. HG.

An elevated GLUT1/GLUT4 ratio, a characteristic feature of cardiac hypertrophy, was found to be significant in cells exposed to high glucose (+137,2%, *P* < 0,01 vs. NG). Interestingly, GLUT1/GLUT4 ratio was significantly reduced in H9c2 exposed to high glucose treated with α-MSH (-55,6%, *P* < 0,01 vs. HG) and with MC5R agonist PG-901 (-56,7, *P* < 0,01 vs. HG). PG-20N, a selective MC5R antagonist, did not change the GLUT1/GLT4 ratio observed in HG cells (Figure 7).

### MC5R Agonism Modulates *in vivo* High Glucose-Induced Cardiac Alterations

As shown in Table 1 by M-mode measurements, diabetic rats (STZ) had a significantly greater left ventricular mass per body weight, compared to non-diabetic rats (+82,6%, *P* < 0,05 vs. CTRL). This was significantly reduced by α-MSH (-30,9%, *P* < 0,05 vs. STZ) and by the doses of 500 and 5000 μg/kg PG-901 (-33,3 and -35,7%, respectively, *P* < 0,05 vs. STZ). While systolic, diastolic and relative wall thicknesses were similar between non-diabetic and diabetic rats, systolic and diastolic left ventricular cavity dimensions were significantly increased in STZ animals, compared to CTRL group (+23,6%, *P* < 0,05 vs. CTRL and +21,6%, *P* < 0,01 vs. CTRL, respectively). Left ventricular internal dimensions in diastole were significantly reduced by MC5R agonists (-13,3% by α-MSH, -13,8% by PG-901 500 μg/kg and -15,1% by PG-901 5000 μg/kg, *P* < 0,05 vs. STZ), as well as the left ventricular internal dimensions in systole (-8,1% by α-MSH, -9,7% by PG-901 500 μg/kg and -12,9% by PG-901 5000 μg/kg, *P* < 0,01 vs. STZ). Diabetic rats showed a significant reduction in left ventricular fractional shortening (LVFS; -26,3%, *P* < 0,01 vs. CTRL), ejection fraction (LVEF; -11,1%, *P* < 0,01 vs. CTRL) and circumferential fiber shortening values (VCF; -28,9%, *P* < 0,01 vs. CTRL), compared to CTRL animals. These values were increased by α-MSH (+22,2% in LVFS,+6,9% in LVEF and +21,9% in VCF, *P* < 0,05 vs. STZ), PG-901 500 μg/kg (+23,8% in LVFS,+7,8% in LVEF and +25,0% in VCF, *P* < 0,05 vs. STZ) and PG-901 5000 μg/kg (+29,2% in LVFS and +28,1% in VCF, *P* < 0,05 vs. STZ;+8,8 in LVEF, *P* < 0,01 vs. STZ). Isovolumetric relaxation time (IVRT) and the ratio of maximal early diastolic peak velocity / late peak velocity of mitral flow (E/A ratio) were significantly decreased in STZ group compared to non-diabetic rats (-26,7 and -20%, respectively, *P* < 0,01 vs. CTRL) and significantly increased by treatment with MC5R agonists (-24,5% in IVRT and -17,4% in E/A with α-MSH, *P* < 0,05 vs. STZ; -26,5% in IVRT and -18,9% in E/A with PG-901 500 μg/kg, *P* < 0,05 vs. STZ; -31,7% in IVRT, *P* < 0,01 vs. STZ and -20,4% in E/A, *P* < 0,05 vs. STZ with PG-901 5000 μg/kg). Myocardial performance index was greater in the diabetic group compared to the non-diabetic animals (+53,6%, *P* < 0,01 vs. CTRL), and it was reduced both by α-MSH administration (-18,6%, *P* < 0,05 vs. STZ ) that by PG-901 at the doses of 500 μg/kg (-20,9%, *P* < 0,05 vs. STZ) and of 5000 μg/kg (-23,2%, *P* < 0,01 vs. STZ). These improved echocardiographic parameters exhibited by diabetic rats treated with MC5R agonists were paralleled also by significantly reduced blood glucose levels (Table 1).

**Table 1:** *In vivo* cardiac parameters evaluated by echocardiography.

	CTRL	STZ	STZ + α-MSH	STZ + PG-901		
				
		65 mg/kg	500 μg/Kg	50 μg/Kg	500 μg/Kg	5000 μg/Kg
Blood glucose (mg/dl)	90 ± 8	351 ± 12^*^	284 ± 9°	331 ± 11	290 ± 13°	274 ± 9°
LV mass/BW (mg/g)	2,3 ± 0,4	4,2 ± 0,5^**^	2,9 ± 0,5°°	3,1 ± 0,4	2,8 ± 0,6°°	2,7 ± 0,5°°
AWTd/TB (cm/cm)	0,42 ± 0,12	0,46 ± 0,11	0,41 ± 0,15	0,43 ± 0,14	0,40 ± 0,10	0,44 ± 0,12
AWTs/TB (cm/cm)	0,76 ± 0,15	0,74 ± 0,12	0,72 ± 0,14	0,75 ± 0,11	0,78 ± 0,14	0,71 ± 0,21
PWTd/TB (cm/cm)	0,41 ± 0,15	0,45 ± 0,18	0,44 ± 0,13	0,42 ± 0,16	0,44 ± 12	0,46 ± 0,11
PWTs/TB (cm/cm)	0,74 ± 0,12	0,71 ± 0,16	0,75 ± 0,22	0,73 ± 0,14	0,75 ± 0,19	0,74 ± 0,18
LVd/TB (cm/cm)	1,82 ± 0,1	2,25 ± 0,1^**^	1,95 ± 0,1°°	1,99 ± 0,2	1,94 ± 0,1°°	1,91 ± 0,1°°
LVs/TB (cm/cm)	0,51 ± 0,008	0,62 ± 0,007^*^	0,57 ± 0,009°	0,61 ± 0,01	0,56 ± 0,01°	0,54 ± 0,008°
RWT	0,41 ± 0,11	0,39 ± 0,09	0,40 ± 0,10	0,42 ± 0,08	0,38 ± 0,11	0,41 ± 0,12
LVFS (%)	44,5 ± 2,5	32,8 ± 2,1^*^	40,1 ± 2,4°°	37,2 ± 2,8	40,6 ± 2,2°°	42,4 ± 2,5°°
LVEF (%)	76,3 ± 1,6	67,8 ± 1,1^*^	72,5 ± 1,8°°	70,7 ± 1,6	73,1 ± 1,4°°	73,8 ± 1,2°
VCF (circ/sec)	0,0045 ± 0.0002	0,0032 ± 0,0003^*^	0,0039 ± 0,0002°°	0,0037 ± 0,0004	0,0040 ± 0,0002°°	0,0041 ± 0,0002°°
IVRT (msec)	41,2 ± 1,52	30,2 ± 1,68^*^	37,6 ± 1,56°°	33,8 ± 1,55	38,2 ± 1,62°	39,8 ± 1,50°
E/A ratio (msec)	1,65 ± 0,05	1,32 ± 0,08^*^	1,55 ± 0,09°°	1,50 ± 0,09	1,57 ± 0,07°°	1,59 ± 0,08°°
MPI	0,28 ± 0,028	0,43 ± 0,016^*^	0,35 ± 0,026°°	0,039 ± 0,028	0,34 ± 0,024°°	0,33 ± 0,019°


LV mass, left ventricular mass; BW, body weight; AWTd, anterior wall thickness in diastole; TB, tibial length; AWTs, anterior wall thickness in systole; PWTd, posterior wall thickness in diastole; PWTs, posterior wall thickness in systole; LVd, left ventricular internal dimensions in diastole; LVs, left ventricular internal dimensions in systole; RWT, relative wall thickness; LVFS, left ventricular fractional shortening; LVEF, left ventricular ejection fraction; VCF, left ventricular circumferential fiber shortening; IRVT, isovolumetric relaxation time; E/A ratio, ratio of maximal early diastolic peak velocity / late peak velocity of mitral flow; MPI, myocardial performance index. Values are expressed as mean ± S.E.M. of *n* = 5 animals; for each rat the average was obtained from three consecutive cardiac cycles. CTRL, non-diabetic rats; STZ, diabetic rats; ^**^P < 0,05 and ^*^P < 0,01 vs. CTRL; °°*P* < 0,01 and °*P* < 0,05 vs. STZ.

## Discussion

Melanocortin receptor 5, (MC5R) predominantly activated by α-MSH and then equally by ACTH, β-MSH, and γ-MSH, is highly expressed in skeletal muscles, exocrine and sebaceous glands, while at a lower level MC5R mRNA is been detected in rodent adipocytes (Chen et al., 1997; Abdel-Malek, 2001; Getting, 2006). Although MC5R functions and signaling are still poorly understood, they are speculated to regulate aldosterone secretion and to mediate neuro-myotrophic, gastric, and anti-inflammatory effects (Gantz et al., 1994; Griffon et al., 1994; Fathi et al., 1995). Interestingly, these G-protein receptors functionally coupled to adenylate cyclase have been recently showed to mediate the skeletal muscle glucose uptake through protein kinase A regulation: the authors demonstrated for the first time that peripheral α-MSH significantly increases glucose uptake via the activation of MC5R and PKA on soleus and gastrocnemius muscles (Abdel-Malek, 2001; Enriori et al., 2016; Hill and Faulkner, 2017).

Although glucose is a primary substrate for heart metabolism and alterations of glucose uptake are tightly associated with cardiovascular diseases, particularly cardiac hypertrophy induced by hyperglycemia, the role of melanocortin receptors in mediating cardiac glucose uptake has been never explored before (Fathi et al., 1995; Wang et al., 2013; Han et al., 2015; Szablewski, 2017). Considering these evidences, we investigated the role of MC5R in mediating cardiac glucose-uptake in H9c2 exposed to high glucose and the role played by MC5R in the glucose-induced hypertrophy. Recent evidences showed that the exposure of rat H9c2 cardiac myocytes to high glucose can be considered an useful *in vitro* model of myocardial hypertrophy, since high glucose levels rapidly induce connective tissue growth factor (CTGF) mRNA that mediates hypertrophy on H9c2 cells (Wang et al., 2009; Han et al., 2015; Li et al., 2017; Wei et al., 2018). Interestingly, H9c2 cells showed a significant up-regulation of MC5R mRNA and protein content following high glucose exposure compared to cells exposed to normal glucose. This increased MC5R expression probably being a defensive and anti-hypertrophic response to the high-glucose induced damage, since by activating MC5Rwith α-MSH or with PG-901 the cell viability was increased and the cardiac hypertrophy markers, including the high intracellular glucose content (detected by ATP levels determination), were decreased compared to cells exposed to high glucose only. Interestingly, the modulation of intracellular glucose content was paralleled by changes in K_ATP_ Inward Rectifier K^+^ Channel 4 (K_IR_6.1 or KCJN8) expression. The *KCNJ8*-encoded Kir6.1 (K_ATP_) channel is an important regulator of vascular tone and cardiac adaptive response to metabolic stress (Tester et al., 2011), activated by low intracellular ATP levels (Takano and Noma, 1993). In our setting, K_IR_6.1 protein levels were significantly decreased by H9c2 exposure to high glucose, in line with previous works (Liang et al., 2016, 2017; Trotta et al., 2017), and were restored by a-MSH and PG-901 treatments. This may be due to the modulation of ATP intracellular levels exerted by MC5R agonists: while the high ATP intracellular content in H9c2 cells exposed to high glucose was paralleled by a decreased K_IR_6.1 protein expression, a reduction in ATP levels was paralleled by an increase in K_IR_6.1 protein levels. Therefore, MC5R stands as a receptor to target in order to modulate the high glucose-induced hypertrophy.

From the molecular point of view and in order to ascertain the intracellular MC5R signaling, our study focused on phosphoinositide 3-kinase (PI3K). This lipid kinase, activated by G protein-coupled receptors (GPCRs) through the direct binding of Gβγ subunits and the small GTPase Ras to PI3K, is a major player for mediating insulin-induced glucose uptake in skeletal and cardiac muscle (Le Marchand-Brustel et al., 1995; Schwindinger and Robishaw, 2001; Riley et al., 2006). Here, we show that both the α-MSH and PG-901 increase the PI3K activity within the H9c2 cells exposed to high glucose, on HEK293 cells (Rodrigues et al., 2009). Moreover, being PI3K also involved in the regulation of H9c2 survival through Akt phosphorylation, leading to a reduction of cell death (Wang et al., 2015; Liu et al., 2017), the protective action of MC5R agonism on cell viability could be just linked to PI3K activation.

PI3K activity is usually modulated by miR-133a, a miRNA involved in regulation of cardiac hypertrophy: increased levels of miR-133aare paralleled by PI3K inactivation (Horie et al., 2009; Abdellatif, 2010; Josse et al., 2014) or viceversa low levels of miR-133a link to PI3K activation. Fitting with this, the high glucose exposure markedly increased miR-133a levels in our setting and reduced PI3K activity. In contrast, the MC5R activation by α-MSH and more selectively with the PG-901 agonist, significantly reduced miR-133a levels, consequently increasing PI3K activity. Moreover, miR-133a knockdown in H9c2 cells exposed to high glucose alone or with MC5R agonists reverted PI3K activity to the levels exposed by normal cells. This confirmed also MC5R modulation of miR-133a levels and consequently, of PI3K activity, evidenced in our experimental setting. However, the molecular mechanisms by which MC5R activation regulates miR-133a levels have to be further investigated.

PI3K inactivation leads to increase of plasma membrane GLUT1/GLUT4 ratio, a feature of pathological cardiac hypertrophy. In a normal adult heart GLUT4 is the primary glucose transporter translocating on plasma membrane after insulin stimulation, while the mediator of basal cardiac glucose uptake GLUT1 is downregulated after birth. Conversely, a pathological hypertrophic condition links a GLUT4 depletion, resulting in a direct increase in GLUT1 levels (Slot et al., 1991; Abel et al., 1999; Paternostro et al., 1999; Tian et al., 2001; Kolwicz and Tian, 2011; Shao and Tian, 2015). PI3K activation appears to be necessary for GLUT4 translocation in the heart, being insulin stimulated GLUT4 translocation blocked by PI3K inhibitor (Egert et al., 1997; Vlavcheski et al., 2018). In line with these evidences, our results furtherly showed that PI3K inactivation induced by high glucose exposure in H9c2 cells lead to a significant increased plasma membrane GLUT1/GLUT4 ratio compared to cells exposed to high glucose. This was reverted by the PI3K activation induced by the stimulation of MC5R with α-MSH and PG-901, thus significantly decreasing GLUT1/GLUT4 ratio.

Therefore, the MC5R is pivotal for cardiac hypertrophy. However, the role exerted by MC5R could have limitations due to the nature of the cells used: H9c2 cells are still physiologically far from being primary cardiomyocytes. On another note, however, these cells presents advantages linked to the fact that they can be easily manipulated and exhibit longer survival and growth with respect to adult cardiomyocytes, that can only be maintained for a short time in culture after a technically challenging isolation (Peter et al., 2016). Moreover, cultures of rat neonatal cardiomyocytes have recently become the standard experimental *in vitro* system used to investigate the aberrant molecular processes occurring during cardiac hypertrophy (Watkins et al., 2011). However, the role of MC5R agonism in modulating cardiac alterations induced by high-glucose was here confirmed also by echocardiographic evaluations in STZ-diabetic Sprague Dawley rats treated with α-MSH and PG-901. The increase shown by STZ-diabetic rats in left ventricular mass per body weight and myocardial performance index, in line with previous evidences (Wichi et al., 2007; Di Filippo et al., 2014), was significantly reduced by α-MSH and PG-901 treatment. Although the increase in LV mass can be interpreted as a consequence of increased myocyte volume and thus hypertrophy it cannot be ruled out that this parameter has changed as a consequence of edema or alterations in the homeostasis of different cell types. Noteworthy, cardiac hypertrophy is the abnormal enlargement, or thickening, of the heart muscle, resulting from increases in cardiomyocyte size and changes in other heart muscle components, such as extracellular matrix. Moreover, the reduced values of left ventricular internal dimensions, fractional shortening, ejection fraction, and circumferential fiber shortening evidenced by STZ-diabetic rats and confirmed by previous works (Joffe et al., 1999; Wichi et al., 2007) clearly evidence a sort of dilated cardiomyopathy, together with a probable fibrosis not measured here, that occurred in these animals, in line with many recent studies in STZ models (Chengji and Xianjin, 2018). These parameters were significantly improved by α-MSH and PG-901, as well as the isovolumetric relaxation time and E/A ratio values.

## Conclusion

**FIGURE 8:**
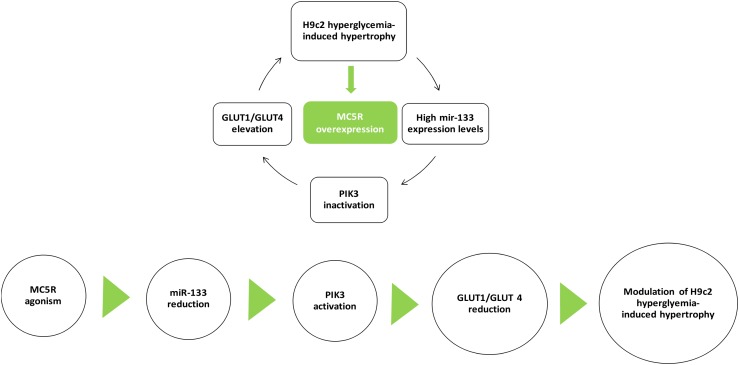
MC5R signaling in cardiac hypertrophy induced by high glucose. H9c2 hypertrophy induced by hyperglycemia is characterized by increased miR-133a levels, leading to PI3K inactivation and GLUT1/GLUT4 elevation. Here, we show for the first time an up-regulation of MC5R, involved in glucose-uptake, as a defensive and anti-hypertrophic response to the high-glucose induced damage. MC5R agonism reduced H9c2 hypertrophic markers by decreasing miR-133a levels, consequently restoring PI3K activity and GLUT1/GLUT4 ratio.

The MC5R seems to be a new target in high glucose-induced cardiac myocytes derangements, and an agonism at this receptor can be a strategic tool to reduce these conditions. *In vitro*, the selective MC5R agonism seems to reduce GLUT1/GLUT4 ratio through PI3K activation, mediated by a decrease in miR-133a levels (Figure 8). These evidences open new possibilities for therapeutic interventions through peripheral melanocortin pathways.

## Author Contributions

MT, RM, and AH performed the research. NA contributed to immunofluorescence analysis and results interpretation. MD analyzed the data. CT and CD designed and wrote the research study.

## Conflict of Interest Statement

The authors declare that the research was conducted in the absence of any commercial or financial relationships that could be construed as a potential conflict of interest.
